# Narrower jugular bulbs and their tributaries are associated with skull base venous hyperintensity on arterial spin-labeling MRI

**DOI:** 10.1038/s41598-026-43549-x

**Published:** 2026-03-08

**Authors:** Periakaruppan V. Manickam, Yusef Qazi, Siddhant Suri Dhawan, Thomas R. Geisbush, Tarik F. Massoud

**Affiliations:** 1 Division of Neuroimaging and Neurointervention, Department of Radiology, Stanford Center for Academic Medicine, Radiology MC: 5659, Stanford University School of Medicine, Radiology MC: 5659, 453 Quarry Road, Stanford, CA 94304 USA; 2 allGood Technology Inc, 764 Channing Ave, Palo Alto, CA 94301 USA

**Keywords:** Jugular veins, Magnetic resonance imaging, Neuroimaging, Skull base, Valsalva maneuver, Anatomy, Diseases, Medical research, Neurology, Neuroscience

## Abstract

Incidental arterial spin-labeling MRI hyperintensity (ASL+) at the jugular bulb (JB) can mimic dural arteriovenous fistula but its anatomical basis remains incompletely understood. We investigated whether JB and tributary sizes are associated with ASL+. We retrospectively analyzed MRIs from 25 ASL+ patients and 25 controls, using post-contrast SPGR images to measure JB and tributary diameters. Group comparisons, effect sizes, false discovery rate (FDR) correction, and receiver operating characteristic (ROC) analyses were performed, with leave-one-out cross-validation (LOOCV) to assess model stability. ASL+ patients demonstrated smaller JB midportion (*P* = 0.047, Cohen’s d = 0.58), JB outflow (*P* = 0.030, d = 0.63), and posterior condylar vein (PCV) diameters (*P* = 0.009, d = 1.02). After FDR correction across eight comparisons, only PCV remained statistically significant. ROC analysis demonstrated moderate discrimination for JB measures (AUC ≈ 0.67) and stronger discrimination for PCV (AUC = 0.78). A multivariate model achieved AUC = 0.83 with stable LOOCV performance (AUC = 0.77), suggesting limited overfitting. Thus, we identify a venous morphometric pattern associated with ASL+, characterized by smaller JB and PCV calibers. However, given multiplicity adjustment and spatial-resolution constraints for PCVs, these associations should be interpreted as exploratory. This composite anatomical configuration may contribute to delayed venous transit and ASL signal trapping, providing a plausible structural contributor to this imaging pitfall and informing future mechanistic investigations.

## Introduction

Arterial spin-labeling (ASL) is a noninvasive MRI technique increasingly adopted in routine clinical practice to provide physiological information that complements structural MRI, and is mainly used to enable in vivo visualization and quantification of regional cerebral blood flow^1,2^. The infrequent demonstration of incidental ASL hyperintense signal (ASL+) at the left jugular bulb (JB) and distal sigmoid sinus often creates a diagnostic dilemma. It is usually ascribed to artifact from “benign jugular venous reflux” in diminutive left internal jugular veins (IJVs)^3–5^. However, this appearance is difficult to distinguish from a less common but potentially dangerous skull base dural arteriovenous fistula (dAVF) requiring additional imaging evaluation. Precise mechanisms for JB venous stasis and consequent ASL+ artifact are not understood fully. Geisbush et al. have recently conjectured a mechanism whereby Valsalva maneuver may possibly reverse the slow flowing blood in the non-dominant left IJV retrogradely towards the skull base; and the consequent reflux of ASL labeled blood past a severe stenosis caused by styloidogenic internal jugular vein compression (SJVC), if present, may contribute to trapping the ASL signal within the JB owing to SJVC-induced venous outlet obstruction^5^.

Given that half of individuals do not have SJVC (43–56%^6^), and severe SJVC stenosis is present in only 18–24%^6^, the presence of left-sided ASL + in many patients requires further critical appraisal and explanation. Intracranial venous outflow primarily occurs through the IJVs, vertebral venous system, and deep cervical veins^7–11^, and is strongly position dependent^8,10,12,13^—in the supine position during patient MRI scanning, the IJV is the dominant drainage pathway. However, venous outflow through the IJVs is influenced by multifactorial conditions^14^ with many interacting variables and determinants that broadly include pressure gradients driving venous flow (inspiration^15^, Valsalva maneuver and Valsalva-like activities^14,16–18^, and conditions with chronically elevated central venous pressure^19–21^; left sidedness and morphological features of IJVs^5^ and brachiocephalic veins^14,22–24^, and other IJV or central venous obstructions at multiple locations from the skull base to the heart^5,6,25–29^; potential collateral pathways^30^; and IJV valve competence^20,21,31–36^.

Clinical emphasis of ASL is placed on identifying cerebral hypoperfusion and hyperperfusion^1^, but with additional consideration of arterial transit artifacts^37^ and high-intensity venous signals when they occur^38^. For the latter, several studies have demonstrated high sensitivity and specificity of ASL for detecting arteriovenous shunt lesions, with reported sensitivity up to 95% and specificity up to 90%^38–42^. This sensitivity reflects the high concentration of labeled blood that bypasses the microvascular bed and is directly delivered into the venous circulation in AVFs and arteriovenous malformations^38–40^. In other scenarios, venous ASL signal may reflect capillary-level shunting rather than a true AVF, potentially indicating reduced oxygen extraction efficiency^43^.

The labeling plane for brain ASL imaging is ideally placed perpendicular to the spine at the C2 or C3 vertebral level^1,44^, which is approximately 4 cm below the cerebellar tonsils, and where both, the internal carotid and vertebral arteries are nearly perpendicular to the ASL labeling plane. This results in high labeling efficiency of arterial blood for both the anterior and posterior intracranial circulations. Simultaneous labeling of venous blood inevitably also occurs in the IJVs but this is dissipated caudally towards the heart. Hence, a recognized pitfall in brain ASL imaging is IJV reflux, in which labeled venous blood ascends from the neck back into the JB and distal intracranial venous sinuses; this then may mimic shunt-related ASL signal from a JB or distal sigmoid sinus dAVF. The presence of reflux-related ASL + at the JB on MRI may therefore reflect the interplay of two mechanisms: retrograde venous reflux that delivers labeled blood to the JB, and structural or flow-related trapping that prolongs label residence and sustains the signal at that location.

Ideally, a comprehensive, integrative approach would be required to elucidate the underlying likely multifactorial mechanisms for left IJV reflux occurrence and how this may consequently generate ASL + at the JB that is specifically related to reflux, and not to a dAVF, in some individuals and not in others. Unfortunately, many of the structural and hemodynamic factors associated with venous reflux and ASL + are difficult to measure concurrently. While a multivariable analysis is ultimately desirable, attempting to interrogate all contributing components within a single study also risks diluting statistical power, introducing collinearity, and obscuring mechanistic interpretation. There are also clear relative merits in establishing the role of individual components in isolation; this can help build a coherent mechanistic framework, particularly in this complex system of factors contributing to IJV reflux and ASL+ where interactions are non-linear and hierarchical. Accordingly, we designed the present study to examine a single aspect of artifactual ASL+, namely to identify a potential structural mechanism for labeled blood trapping within the left JB related to skull base anatomical features visible on routine brain MRI at the same time ASL + is observed, independent of additional venous imaging of the neck or chest that could be obtained separately. This reductionist approach allows for more precise hypothesis testing, clearer attribution of observed effects, and more robust interpretation of results.

We here hypothesize that in the absence of any substantial SJVC, obstruction in the venous outflow of left JBs and their tributaries owing to unfavorably narrow morphologies might similarly predispose to blood trapping, stasis, and consequent ASL+. We therefore investigate this potentially novel association between vascular narrowing in left JBs plus their tributaries and incidental ASL + by performing an MRI morphometric comparison of these structures in patients with and without this imaging artifact. We demonstrate that smaller JB and posterior condylar vein (PCV) diameters are associated with ASL+, suggesting a venous morphometric pattern that may contribute to this imaging finding and merits further prospective validation.

## Results

**Fig. 1:**
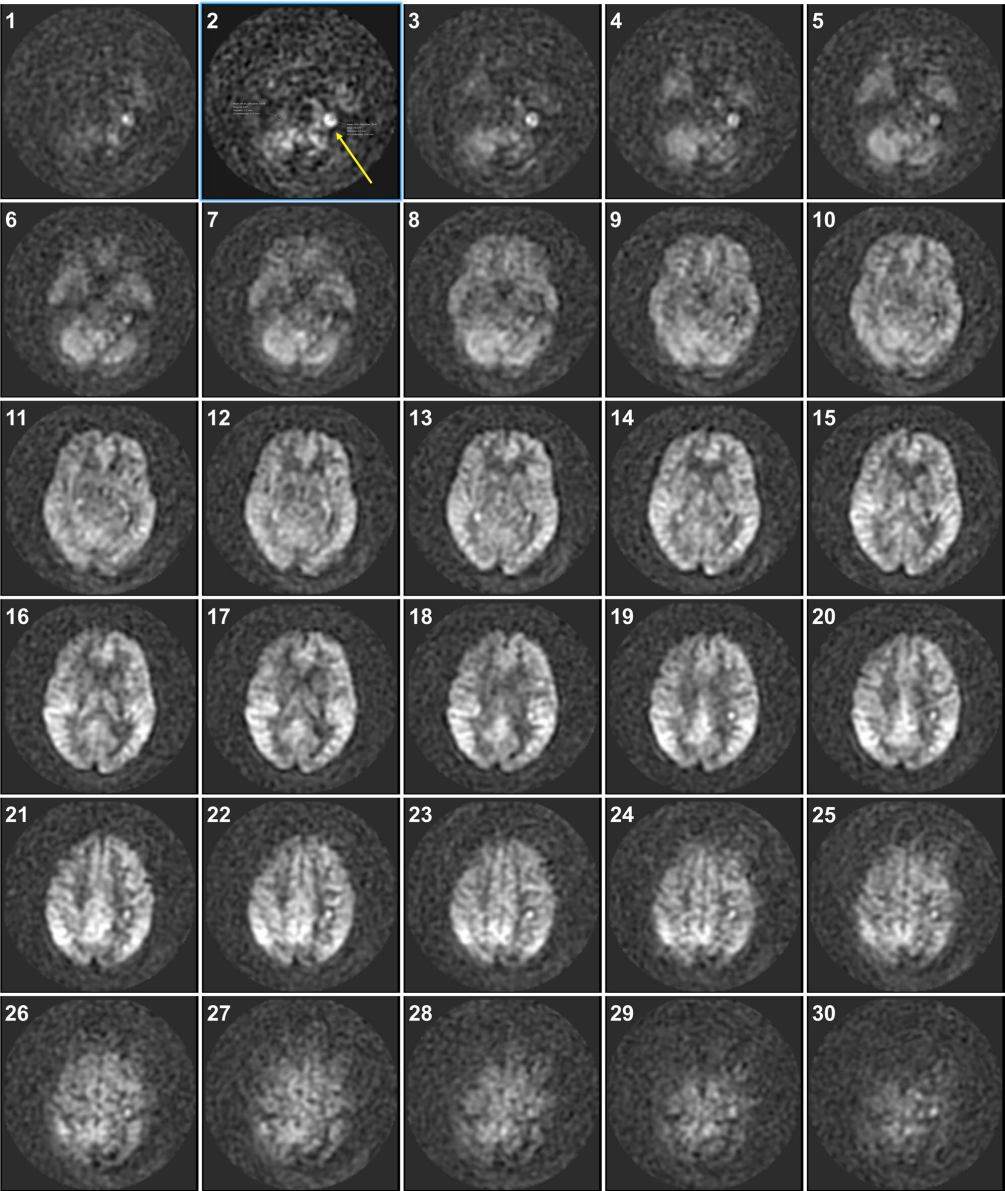
Appearances of all axial slices for an ASL-MRI study on an 80-year-old female showing ASL hyperintensity at the left skull base. Caudad images through the skull base start at image 1 and successive craniad sections end at the vertex at image 30. Successive images 1 to 6 show a focal left-sided skull base hyperintensity with the strongest signal seen on image 2 (arrow).

**Fig. 2:**
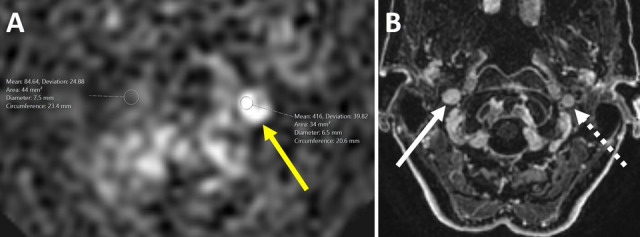
Appearances of ASL + and a corresponding diminutive JB. **A** Magnified view of the focal left-sided skull base hyperintensity on an axial ASL-MRI image in the same study for an 80-year-old female shown in Fig. 1. For illustrative purposes, and using the images of this patient as an example, we drew a region of interest that shows increased quantitative signal intensity in the area of the left JB (yellow arrow) compared to the signal in the right JB. **B** Axial post contrast SPGR image from the same patient shows the location of this ASL hyperintensity corresponds to a diminutive left JB (dashed arrow pointing to JB outflow segment). By comparison, solid arrow shows the dominant right JB.

Patient age distributions and means and standard deviations for all measured parameters are shown in Table 1. There were equal numbers of males and females across both groups, and mean age of all participants was 68.2 ± 13.3 years. All ASL+ patients had ASL hyperintensity at the left skull base (Figs. 1 and 2A) localized to the left JB with or without distal sigmoid sinus involvement—none were right-sided. All IJVs in both patient cohorts were diminutive on the left (Fig. 2B) when compared to dominant on the right because we had selected our CSs to have diminutive left IJVs that matched a similar finding in all ASL+ patients. We did this to avoid any inherent variation in relative dominance of right or left IJVs that might act as a confounder when comparing our measured JB morphometrics in both cohorts (see Methods).

**Table 1:** Demographics and morphometric characteristics of JBs and their venous tributaries for all study participants (CSs and ASL+ patients), and statistical correlations of these characteristics between the two cohorts.

Characteristic	Control Subjects *n* = 25 mean, (SD, 95% CI)	ASL+ Patients *n* = 25 mean, (SD, 95% CI)	Statistical Significance of ASL + vs. CSs
Age of patients (years)	67.6 (12.8, ± 5.3)	68.8 (14.1, ± 5.8)	NS
JB inflow AP diameter (mm)	6.8 (1.8, ± 0.7)	6.5 (2.0, ± 0.8)	NS
JB midpoint AP diameter (mm)	11.0 (3.6, ± 1.5)	9.1 (2.9, ± 1.2)	*P* < 0.047
JB outflow AP diameter (mm)	9.6 (3.1, ± 1.3)	7.9 (2.5, ± 1.0)	*P* < 0.030
JB volume (mm ^3^ )	797.6 (390.0, ± 157.5)	625.2 (430.4, ± 177.7)	NS
JB cross sectional area (mm ^2^ )	73.7 (22.2, ± 9.2)	72.2 (36.0, ± 14.9)	NS
Sigmoid sinus maximum diameter (mm)	6.0 (1.3, ± 0.54)	5.7 (1.2, ± 0.5)	NS
Inferior petrosal vein maximum diameter (mm)	4.1 (0.9, ± 0.36)	3.7 (1.0, ± 0.4)	NS
Posterior condylar vein maximum diameter (mm)	2.9 (0.9, ± 0.38)	2.1 (0.6, ± 0.3)	*P* < 0.009

*ASL +* patients with focal left-sided skull base ASL hyperintensity, *AP* anteroposterior, *CI* confidence interval, *CS* control subject, *NS* not significant, *SD* standard deviation.

**Fig. 3:**
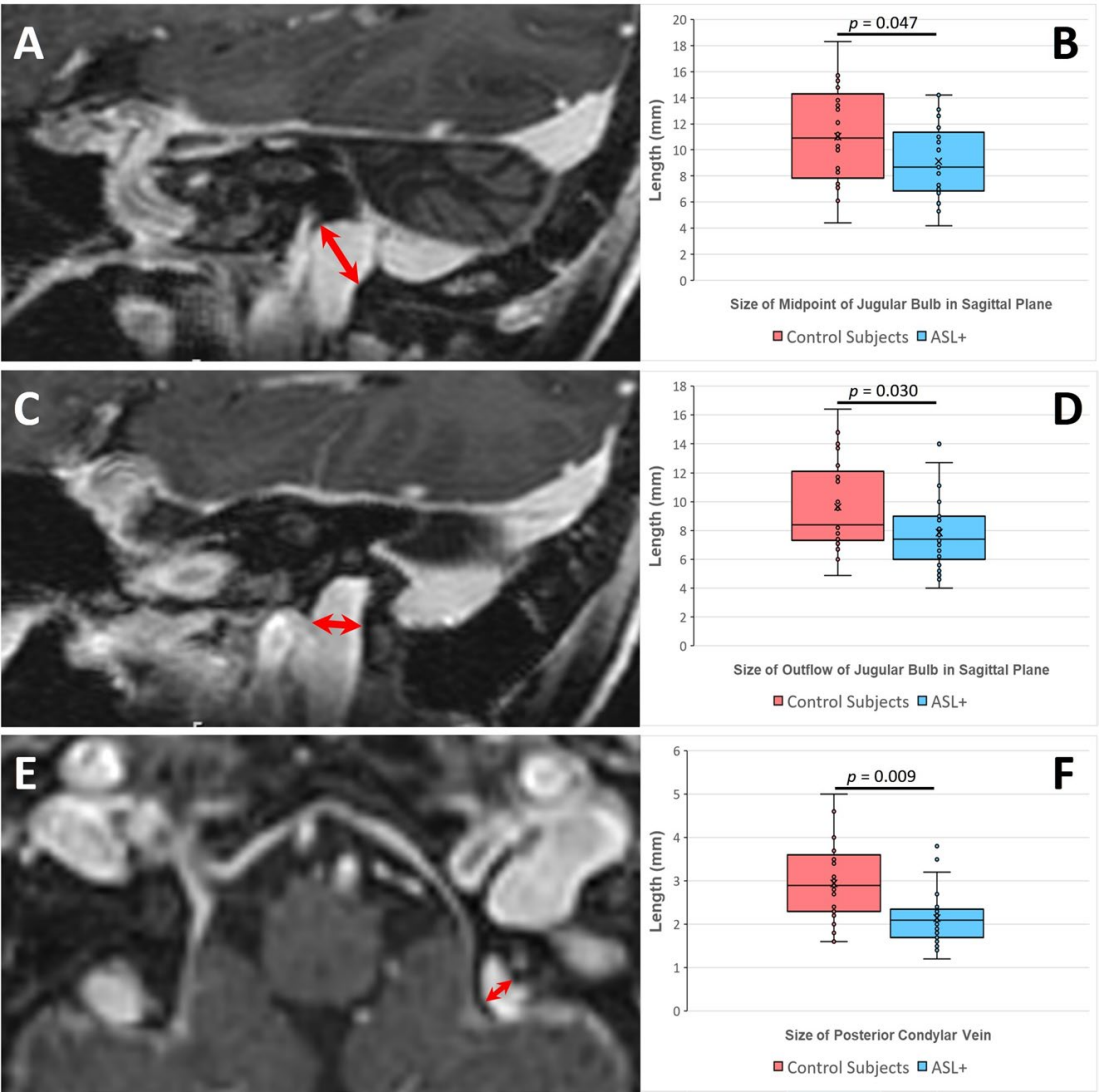
Morphometric features of left JBs and their tributaries on multiplanar post contrast SPGR images. Exemplified images are shown for the same 80-year-old female as in Fig. 1. **A** Diameter of the JB midportion (red dimension line) viewed parasagittally, and **B** box and whiskers plots show significantly smaller mean diameters in ASL+ than in CSs. **C** Diameter of the narrowest point of JB outflow (red dimension line) viewed parasagittally, and **D** box and whiskers plots show significantly smaller mean diameters in ASL+ than in CSs. **E** Diameter of the posterior condylar vein (red dimension line) viewed axially, and **f** box and whiskers plots show significantly smaller mean diameters in ASL+ than in CSs.

Importantly, all morphometric features recorded for JBs and tributaries were smaller in ASL+ than in CSs (Table 1). Additionally, the mean diameter of the JB midportion when viewed parasagittally (*P* = 0.047, Cohen’s d = 0.58) (Fig. 3A, B), mean diameter of the narrowest point of JB outflow when viewed parasagittally (*P* = 0.030, Cohen’s d = 0.63) (Fig. 3C, D), and mean diameter of PCVs (*P* = 0.009, Cohen’s d = 1.02) (Fig. 3E, F) were all significantly smaller in ASL+ than in CSs. Thus, effect size analysis demonstrated moderate between-group differences for JB midportion and JB outflow (d = 0.58 and 0.63, respectively) and a large effect size for PCV diameter (d = 1.02). There were no other significant differences or correlations, including with patient age and sex. There was a similar prevalence of JB diverticula in both groups (five in ASL + and four in CSs).

After Benjamini-Hochberg false discovery rate (FDR) correction across eight comparisons (q = 0.05), only PCV retained significance (adjusted *P* = 0.006), while JB outflow (adjusted *P* = 0.121) and JB midportion (adjusted *P* = 0.124) did not. Permutation testing (10,000 permutations) confirmed this pattern (PCV corrected *P* = 0.009; JB outflow *P* = 0.232; and JB midportion *P* = 0.330).

**Fig. 4:**
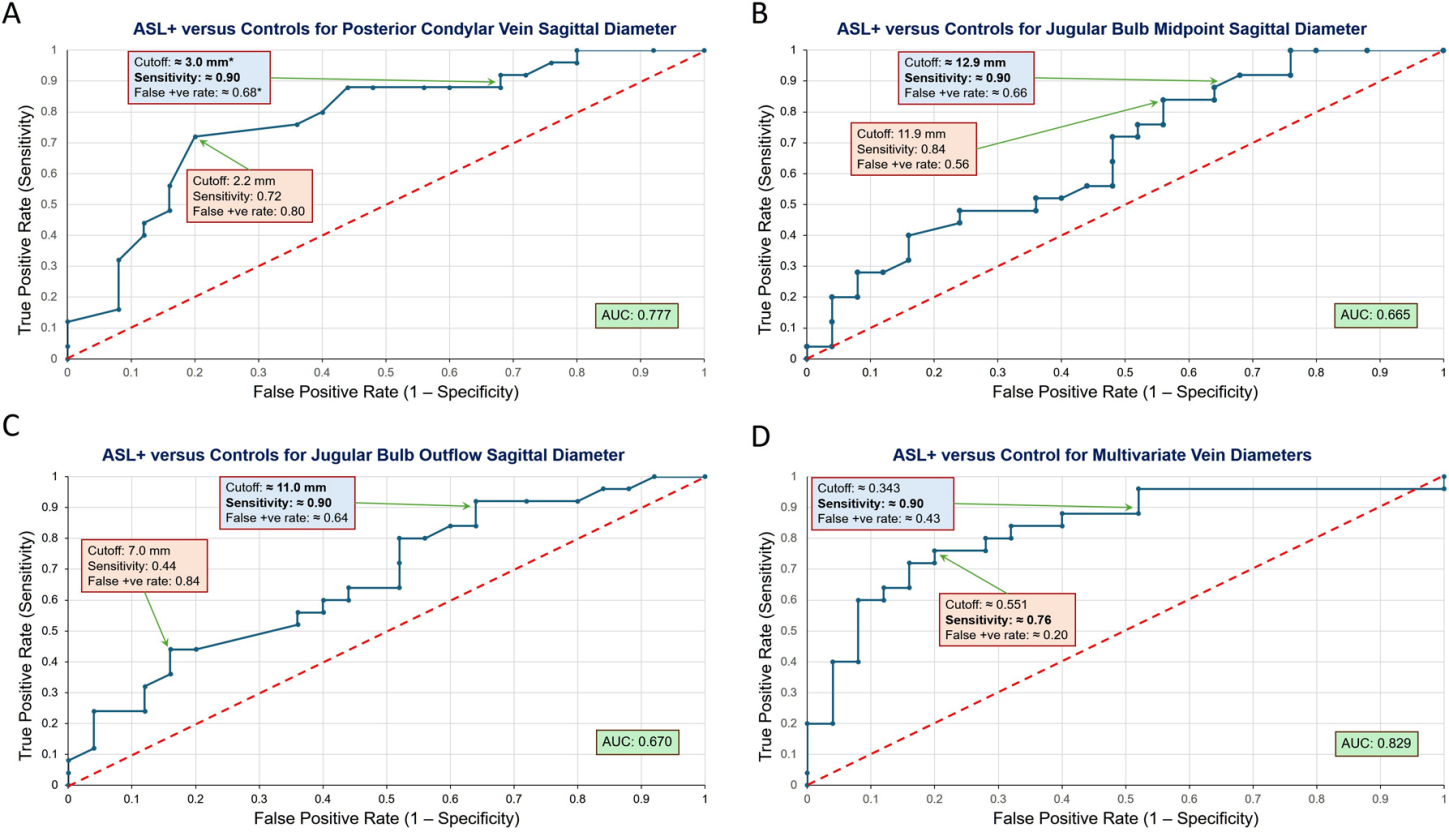
Receiver-operating-characteristic (ROC) curve analysis to distinguish ASL+ from control cases using JB and PCV dimensions as potential imaging biomarkers. We evaluated a univariate logistic regression statistical model to distinguish presence of ASL+ using the mean diameter of: **A** PCVs, **B** JB midportion, and **C** narrowest point of JB outflow. We also performed ROC analysis using multivariate logistic regression to evaluate the discriminatory potential of combining all three venous dimensions (**D**). Optimal sensitivity, specificity, and cutoff values according to Youden’s index are shown in pink boxes, and calculated similar values that yield a 90% sensitivity are shown in blue boxes. AUC values are in green boxes. Asterisk* denotes an estimate within a small range.

Analysis of receiver-operating-characteristic (ROC) curves showed that mean diameter of PCVs (an area-under-curve [AUC] of 0.78; leave-one-out cross-validation [LOOCV] AUC of 0.75) (Fig. 4A) was the best metric to distinguish ASL+ from CSs with a cutoff value of 2.2 mm, although this finding should be considered exploratory given that PCV caliber approaches the spatial resolution limit of our imaging. The mean diameter of the JB midportion when viewed parasagittally (an AUC of 0.67; LOOCV AUC of 0.58, with a cutoff of 11.9 mm) (Fig. 4B), and mean diameter of the narrowest point of JB outflow when viewed parasagittally (an AUC of 0.67; LOOCV AUC of 0.60, with a cutoff of 7 mm) (Fig. 4C) showed lower discriminative ability that degraded further under cross-validation, suggesting that these individual metrics are less stable than PCV and have more modest standalone discriminatory performance. Sensitivities and specificities at cutoff values based on Youden’s index, as well as calculated specificities and cutoff values that theoretically would yield sensitivities of 90% (that is, what would be optimal for a diagnostic biomarker) are also shown in Fig. 4A-C). For the univariate analysis, any diameter below the cutoff would identify ASL+ status.

The multivariate ROC analysis of the combined venous measurements produced an AUC of 0.83 (optimal sensitivity of 0.76 and specificity of 0.80, and an optimal probability threshold of 0.551) (Fig. 4D). Thus, the multivariate approach produced a model that effectively discriminated between ASL+ patients and CSs, outperforming individual ROC analyses. LOOCV of the multivariate model yielded an AUC of 0.77, representing a slight reduction from the original AUC of 0.83 and indicating stable generalizability despite the limited sample size. A DeLong’s test confirmed statistical differences of AUC values for the multivariate combined measurements compared with the JB midportion alone (*P* = 0.025), and with the JB outflow alone (*P* = 0.022). However, there was no significant difference of AUC values between the combined measurements and that of the PCV (*P* = 0.268) because the standalone AUC for PCV was already high at 0.78. Importantly, when using this multivariate approach, the cutoff value is not a single 3-dimensional point representing a composite of three venous measurements, but rather an infinite set of 3-dimensional points. For example, we calculate that for the same optimal probability threshold of 0.551, three different venous measurements (for the mean diameter of the JB midportion, mean diameter of the narrowest point of JB outflow, and mean diameter of PCVs, respectively) might be: (1) 16.82 mm, 5.84 mm, and 2.78 mm; (2) 13.45 mm, 2.94 mm, and 3.36 mm; and (3) 10.08 mm, 14.55 mm, and 1.28 mm. Our model shows that a decrease in any of the three venous diameters increases the likelihood of ASL+, with the PCV diameter showing the strongest association, though this association warrants validation using higher-resolution imaging given its proximity to the spatial resolution limit. At an exploratory 90% sensitivity threshold, the model achieved an interpolated specificity of 0.57 at a probability cutoff of 0.343. While this specificity is modest, the primary use of these thresholds would be to provide supportive evidence when evaluating incidental ASL+ rather than serving as standalone diagnostic criteria. Unlike the univariate analysis, a higher probability threshold distinguishes ASL+, which is supported by the negative correlation coefficients in our logistic regression.

## Discussion

In this study we aim to build on the previous imaging investigations of Caton et al.^4^ and Geisbush et al.^5^ that seek explanations for the phenomenon of incidental artifactual ASL + at the left skull base. This greater knowledge would help to address this neuroimaging pitfall with greater confidence and potentially lessen the need for further imaging directed at diagnostic exclusion of a dAVF.

This study demonstrates consistent venous morphometric differences between ASL+ patients and anatomically matched controls, characterized by smaller JB midportion, JB outflow, and PCV diameters in the ASL+ cohort. Effect size analysis revealed moderate anatomical separation for JB parameters (Cohen’s d = 0.58–0.63) and a large separation for PCV (d = 1.02), supporting the presence of a measurable venous anatomical pattern associated with ASL+. However, PCV caliber in the ASL+ cohort approaches the effective spatial resolution of 0.9 mm for isotropic post-contrast SPGR imaging and is therefore more susceptible to partial-volume and plane-selection effects. Diameter differences on the order of approximately one voxel warrant cautious interpretation, particularly when proposing threshold values. Accordingly, our PCV findings—and any ROC-derived cutoff—should be considered exploratory pending validation using higher-resolution venous imaging and/or cross-sectional area–based measurements in planes perpendicular to vessel centerlines.

Conversely, JB midportion and outflow diameters are substantially larger than voxel dimensions and therefore less susceptible to partial-volume effects. Their measurement robustness supports the biological plausibility of JB morphometric contributions to ASL+. However, multiplicity correction using the Benjamini–Hochberg procedure resulted in loss of formal statistical significance for JB midportion and JB outflow differences. On the other hand, these dimensions demonstrated moderate effect sizes and consistent directional differences, suggesting that limited sample size and conservative correction across correlated anatomical measures may have reduced statistical power. In contrast, PCV diameter demonstrated a large effect size and retained FDR significance. Although only PCV diameter retained statistical significance after FDR correction, the directionality of differences was consistent across all measured venous structures, suggesting a coherent morphometric trend rather than an isolated finding. Indeed, the multivariate model incorporating all three parameters demonstrated stable performance under cross-validation, with only modest degradation (AUC 0.83 to 0.77), supporting the interpretation that ASL + may reflect a composite venous morphometric configuration rather than a single dominant anatomical discriminator.

Importantly, effect size magnitude and cross-validation stability provide complementary information beyond P values alone. The moderate effect sizes observed for JB measures may reflect underpowered but potentially real signals rather than statistical noise, and the stability of the multivariate model under LOOCV suggest that these anatomical differences are unlikely to represent random variation, though prospective validation is required. Taken together, our findings support the hypothesis that ASL+ localized to the JB may reflect a composite venous morphometric environment characterized by relative narrowing of outflow pathways and reduced collateral venous caliber. However, given the exploratory nature of these findings after multiplicity correction and resolution constraints, these results should be interpreted as hypothesis-generating rather than confirmatory. Any derived thresholds should not be considered prescriptive for clinical decision-making at this stage.

Based on these preliminary findings and pending future more rigorous validation studies, we speculate that JBs with significantly narrower midportions and smaller outflows into the IJVs and PCVs may possess configurations that favor trapping, stasis, and recirculation of ASL-labeled venous blood. We did not intend in this investigation to establish definitive causality, as this would involve a more complex study requiring multivariable analysis of several potential factors at play. Therefore, within the context of this exploratory study, we believe that our observations are important because, to our knowledge, no previous reports have highlighted such a potential association. Hence, it is conceivable that a narrow JB morphology may be an additional contributory factor visible on routine brain MRI (which usually incorporates images of the skull base and upper neck) that would explain the origin of ASL + in a manner similar to that described previously for trapping of ASL label by severe SJVC. Additionally, a future confirmation of significant narrowing of PCVs in ASL+ patients would add to any such venous stasis by diminishing JB outflow or inflow via these bidirectional emissary veins.

Our primary aim was to further understand possible mechanisms at the root of this ASL diagnostic pitfall, which often proves challenging in clinical practice. The overall practical course of action for a neuroradiologist to distinguish this artifact from similar and accompanying MRI findings of a skull base dAVF, and the rationale for any additional imaging – which is not always necessary – must also be considered^4,5^.

Intracranial dAVFs account for approximately 10–15% of intracranial vascular malformations and arise from abnormal shunts between meningeal arteries and dural venous sinuses or cortical veins^45–47^. They most commonly occur in the cavernous sinuses or at the transverse–sigmoid sinus junction where venous drainage is typically directed toward the ipsilateral JB and IJV or, less frequently, the contralateral transverse-sigmoid sinus and jugular system^47^. Most dAVFs behave benignly^46^; lesions without cortical venous drainage (Cognard types I and IIa) carry very low annual risks of neurological events and mortality and may even involute spontaneously^48^. Benign fistulae are more prevalent than aggressive forms^47^, but incidental detection—particularly in transverse-sigmoid sinus dAVFs—is quite uncommon^47^.

The clinical concern regarding ASL + at the left jugular bulb and distal sigmoid sinus lies in the possibility that it reflects an unrecognized dAVF with cortical venous drainage, which confers substantial risks of hemorrhage, neurological deficit, and death^46^. Moreover, conversion of benign dAVFs to aggressive forms can occur but is typically associated with new or worsening neurological symptoms^49^. Notably, none of our patients demonstrated clinical features commonly associated with transverse–sigmoid sinus dAVFs, such as pulsatile tinnitus, cranial bruit, or persistent headache^46,47^, substantially lowering the pretest probability of clinically significant shunting.

As digital subtraction angiography (DSA) is the gold standard for dAVF diagnosis^46^, a key clinical question is whether all patients with incidental ASL + at the left JB and distal sigmoid sinus should undergo angiographic evaluation, particularly in the absence of clinical stigmata and given the very low incidence of incidentally discovered dAVFs. An alternative approach is to assess whether secondary or circumstantial imaging evidence on routine MRI sequences, with or without additional cross-sectional imaging (CT angiography [CTA] or MRI-based), can help distinguish benign IJV reflux from dAVF^50^. As such, MRI provides valuable noninvasive assessment of dAVFs through identification of flow voids, venous ectasia, parenchymal enhancement, abnormal venous morphology, and increased vascularity of adjacent arterial branches recruited to a fistula^4,46,50^. When uncertainty remains, noninvasive adjuncts—including multi-PLD ASL^1^, TOF-MRA^4,51^, or time-resolved contrast-enhanced MRA^4,52^—may help differentiate reflux-related ASL signal from true arteriovenous shunting and guide the need for angiographic evaluation.

A conceptually ideal study design would involve direct comparison of JB morphometrics among patients with ASL+, stratified by the presence or absence of a dAVF, along with a control group with absent ASL+. However, such a design is difficult to implement in practice because ASL + is uncommon and intracranial dAVFs at the JB and sigmoid sinus are rare, substantially limiting the number of eligible patients and the statistical power of such comparisons. As a pragmatic alternative, all our study patients were preselected using criteria that excluded a prior history or clinical presentation suggestive of dAVF so as to focus on patients with ASL+ owing to benign IJV reflux and with a high likelihood of being unconfounded by the presence of a dAVF. At the time of ASL+ detection, none of the patients demonstrated concurrent or subsequent MRI, CTA, or DSA findings, nor developed clinical symptoms indicative of a dAVF.

Based on a thorough statistical analysis of dimensions for JBs and their tributaries, our secondary aim in this study was to explore the generation of additional practical clinical guidance when encountering ASL+. Importantly, for the reasons discussed earlier, any derived dimensional thresholds in this exploratory study should not be considered prescriptive for clinical decision-making at this stage. Pending confirmation in future more rigorous mechanistic studies, it is conceivable we could discriminate ASL+ from control cases in a manner similar to using the logistic regression equation described in the Methods and by comparing it to a desired level of sensitivity/specificity. More information regarding thresholds for different sensitivity and specificity levels when evaluating all three venous dimensions together can be found at: https://asl-roc.streamlit.app.

There are only a few literature accounts of morphological variations in sizes and shapes of JBs and their tributaries^53–55^. An important additional consideration and potential confounder pertinent to our study is whether the detected JB and PCV narrowing in the presence of ASL + may reflect a physiological change in vessel dimension secondary to drainage from dAVFs into arterialized JBs. The presence of stenotic arterialized veins draining arteriovenous shunts is a well understood hemodynamic phenomenon reactive to prolonged increase in flow, pressure, and wall shear stress on veins^56^. For instance, bilateral JB stenosis has been studied in children with high-shunting vein of Galen malformations^57^. This is another reason why it would be important to ensure the absence of stenosis-inducing dAVFs when determining potential mechanisms for ASL+ development owing to benign IJV reflux rather than from a shunting dAVF. In this retrospective study, and in the absence of impractical DSA studies on all patients, we logically inferred a very low likelihood of a dAVF by finding no secondary imaging or clinical evidence of shunting on contemporaneous or follow-up cross-sectional imaging.

Hence, we assume that the constrictions in JBs we recorded in the ASL+ cohort are congenital in origin rather than associated with other diseases such as craniosynostosis, where patients have significantly smaller jugular foramina^58^, or patients with sclerosteosis, a rare hyperostotic disorder^59^. Both conditions can lead to marked JB stenoses and consequent raised intracranial pressure. Notably, the mean JB diameters for all our study participants were in the 5 mm to 15 mm range, previously and arbitrarily considered by Tudose et al. as normative values^60^. Thus, we emphasize that in addition to the overall sizes of JBs being smaller in ASL+ patients, the more significant differences we recorded for JB midpoint and outflow diameters should be considered as relatively focal narrowings rather than full stenoses in the JBs.

There are several limitations to this exploratory study including its retrospective retrieval of data and lack of structural and functional correlations with multiple other potential or known factors that contribute to IJV reflux, and which would be necessary in future rigorous multifactorial evaluations to determine the cause of ASL+. Indeed, we view this study as foundational rather than exhaustive. By rigorously characterizing one component, the findings can be integrated into future investigations that expand toward multivariable or systems-level models of intracranial venous outflow to study ASL+. Such an incremental strategy is widely adopted in the study of complex physiological scenarios and diseases, and is essential for generating interpretable, reproducible insights that ultimately inform broader integrative analyses.

As initial case identification for our study relied in part on report terminology, some ASL+ cases may not have been captured during screening. However, all included cases were subsequently confirmed by direct review of ASL images using predefined imaging criteria, ensuring consistent classification. Consequently, any limitation in case capture rather than case classification would be unlikely to systematically influence the observed anatomical associations. Future prospective studies incorporating systematic imaging-based review of consecutive ASL examinations, independent of report terminology, would enable more complete case ascertainment and allow complementary evaluation of incidence, anatomical variability, and hemodynamic correlates. The present study was instead designed as a focused mechanistic investigation of anatomical associations within a rigorously confirmed cohort.

In routine neuroradiological practice, DSA is not performed in asymptomatic patients lacking clinical or cross-sectional imaging features suggestive of dAVF, reflecting a low pretest probability and procedural risk-benefit considerations. As systematic angiographic confirmation was not performed in most of our retrospective patients, the presence of rare occult low-grade fistulas cannot be excluded entirely; however, the absence of clinical or other imaging features of arteriovenous shunting indicates a decidedly low likelihood of dAVF, and future prospective studies incorporating systematic angiographic or hemodynamic validation would further strengthen mechanistic interpretation.

There are also several limitations regarding statistical interpretation and image resolution considerations, as discussed earlier. First, morphometric findings did not uniformly retain statistical significance after FDR correction, and thus should be interpreted cautiously. Second, PCV measurements in the ASL+ cohort approached the spatial resolution limit for our SPGR images and may have been influenced by partial-volume effects. Third, cross-validation, while mitigating overfitting concerns, does not replace independent external validation (across scanners, protocols, and centers) that accounts for sampling bias, overfitting to local characteristics, measurement differences, and patient population differences. Future prospective studies incorporating larger cohorts, higher-resolution venous imaging, independent blinded measurements, and comprehensive hemodynamic assessment will be necessary to confirm and refine these anatomical associations.

Importantly, in addition to the JB narrowing addressed in this study, and SJVC investigated by Geisbush et al. previously^5^, the other causes discussed earlier for venous flow obstruction that delay or reverse blood flow in the IJVs would also need to be considered in future multivariable analysis. In particular, future studies incorporating comprehensive hemodynamic assessment—including reflux direction, venous flow velocity, pressure gradients, and multi-delay ASL acquisitions—will be important to better characterize venous flow dynamics and distinguish true venous residence effects from labeling arrival or timing-dependent ASL behavior. This is particularly relevant given that single-PLD ASL limits physiological interpretation and that venous signal may be strongly state-dependent, including effects related to respiration, Valsalva-like maneuvers, and central venous pressure. Additionally, the role of left brachiocephalic vein compression warrants further evaluation. It is known that deep inspiration increases the distance between the sternum and thoracic outlet arteries, to enhance venous drainage of the left IJV into the left brachiocephalic vein^15^. However, if a persistent and sufficiently high reversed pressure gradient is present, such as with central venous obstruction, continuous IJV reflux may occur^14,23,61^. This reflux is reported predominantly on the left side owing to the longer, more oblique course of the left brachiocephalic vein and its passage through the narrow anterior portion of the superior mediastinum, where compression can lead to stenosis or occlusion^14^. This phenomenon is uncommon in healthy individuals^62,63^ but occurs more frequently in the elderly, likely due to age-related vascular changes^23^.

In our study, ASL+ classification was based on standardized qualitative anatomical criteria rather than quantitative signal intensity measurements. Future studies incorporating quantitative ASL signal analysis, assessment of the extent of venous involvement (JB alone versus extension into the sigmoid sinus), as well as the comprehensive anatomical and hemodynamic parameters mentioned earlier will be important to enable multivariable analysis and provide a more comprehensive characterization of the anatomical and physiological determinants of ASL+. In addition, as measurements in our study were performed by consensus rather than independent blinded readers, formal inter-observer reproducibility was not separately quantified. Future studies incorporating independent blinded measurements would further validate reproducibility and generalizability of the proposed morphometric thresholds.

Another possible confounding factor is based on the observation by Wang et al. that the size of the left JB, but curiously not the right one, decreases significantly with age^64^. Therefore, future prospective studies using larger patient cohorts and with age stratification are needed to clarify the role of both structural and functional characteristics of JBs and IJVs, along with other etiologic factors that may contribute to diagnostically-challenging incidental ASL + at the left skull base.

## Conclusion

This study demonstrates consistent venous morphometric differences associated with artifactual ASL signal hyperintensity at the left JB, including smaller diameters of the JB midportion and outflow, and of PCVs, with moderate-to-large effect sizes suggesting a coherent venous anatomical pattern. While only PCV caliber retained statistical significance after multiplicity correction—and warrants cautious interpretation given its proximity to imaging spatial resolution—the overall findings support the concept that ASL + may reflect a composite venous morphometric configuration contributing to delayed venous transit and trapping of ASL signal. These associations should be considered hypothesis-generating and not prescriptive for clinical decision-making at present; however, in the absence of clinical or imaging features suggestive of a dAVF, such anatomical features may help contextualize ASL + as a benign venous phenomenon. Our findings will inspire future prospective studies incorporating larger cohorts, higher-resolution venous imaging, independent validation, and comprehensive hemodynamic assessment that will be critical to fully elucidate the anatomical and physiological determinants of ASL + and their clinical significance.

## Methods

We obtained IRB ethical approval, which included waiver of individual authorization for recruitment and informed consent pursuant to HIPAA information in the protocol application. This study was designed as a focused mechanistic analysis of anatomical associations of ASL+ within its characteristic venous anatomical context, rather than an epidemiologic or hemodynamic evaluation of unselected ASL+ populations.

### Patient selection

In this retrospective case-control study, we used our institutional database search engine to retrieve radiological reports for adult patients accrued over a 7-year period (August 2015 to October 2022) after MRI evaluation of various neurological symptoms. For this, we applied inclusion criteria (presence in the report of keyword combinations of “ASL” and “reflux” [when raised by the radiologist as a diagnostic consideration], with either “jugular bulb” or “skull base”, which we found in 236 reports) and exclusion criteria (mentions of a known dAVF involving the left skull base, clinical stigmata (tinnitus, bruit, headache) suggesting or raising suspicion of a possible dAVF at the skull base, or known intracranial or neck conditions that can alter venous hemodynamics, all found in 8 reports among the 236 reports). Most of the “ASL” mentions in the resulting 288 reports were spurious hits owing to its use with the other keywords in contexts unrelated to the subject of ASL+. We thus selected 30 reports initially reflecting eligibility of 30 ASL+ patients, but after subsequent analysis of their post-contrast SPGR volumetric imaging findings we excluded five additional patients who had severe SJVC, defined by Geisbush et al. as ≥ 75% IJV stenosis^5^. Thus, we identified 25 patients for our ASL+ group. In these patients, their MRI indications were for evaluation of brain metastases (6 patients), cerebrovascular events (6), motor weakness (3), small frontal lobe mass (2), follow-up of treated aneurysms (2), head trauma (1), memory loss (1), neck and eye pain (1), nasopharyngeal mass (1), questionable meningitis (1), and transient headache (1). We then randomly selected 25 age and sex-matched patients, similarly with no clinical indicators of a possible dAVF, as control subjects (CSs) without ASL+. Note that right-sided ASL + is rare, and we therefore anticipated focusing our analysis on left-sided IJVs and JBs. Accordingly, based on initial review of the ASL+ cohort (see Results), the 25 patients acting as CSs were all intentionally selected to have diminutive left IJVs, mirroring the morphology observed in all ASL+ patients. Consequently, both cohorts exhibited smaller left IJVs relative to right-sided dominance. This design controlled for variation in IJV dominance that could otherwise confound comparative JB morphometric analysis. Importantly, CSs were anatomically matched to reflect the venous morphology in which ASL+ occurs, allowing isolation of JB morphometric differences within the relevant anatomical context rather than across heterogeneous dominance patterns unlikely to contribute to ASL+. On imaging, all patients had otherwise normal or incidental intracranial and neck findings not pertinent to the study question without conditions that might alter venous hemodynamics.

For the 25 patients with ASL+, we also retrospectively reviewed the other MRI sequences obtained concurrently and subsequently at follow-up to search for: (1) whether source and maximum intensity projection (MIP) images of any 3D time-of-flight magnetic resonance angiography (MRA) showed additional evidence to support the diagnosis of a dAVF (increased vascularity from branches of the external carotid artery [ECA] plus venous flow-related enhancement in the JB and distal sigmoid sinus) versus IJV reflux alone (increased venous flow-related enhancement in the JB and distal sigmoid sinus without prominence of ECA branches); and (2) if any subsequent CT angiography (CTA) or invasive digital subtraction angiography (DSA) was performed to confirm or exclude a dAVF. This retrospective analysis of the imaging studies performed for our 25 ASL+ patients showed: 10 patients had a concurrent MRA; eight of these patients showed venous flow-related enhancement, and two did not. None had increased ECA vascularity. Of the remaining 15 patients, 13 did not have a subsequent follow-up MRA, and only two did, showing venous flow-related enhancement but no increased ECA vascularity. One of the patients had a follow-up CTA that was normal. By chance, only 2 patients had follow-up invasive DSA studies, both showing no dAVF, for clinical questions unrelated to ASL+, i.e. work-up of brain aneurysms.

### Imaging parameters

We obtained all MRI scans on 3 T MR units (Discovery MR450, Optima MR450, or Signa HDx, all GE Medical Systems) using standard head coils with 8 elements (8 h Brain; GE Healthcare). Images were acquired using the same standardized brain protocol that included multiplanar volumetric post-contrast SPGR sequences using conventional imaging parameters (echo time = 3 ms, repetition time = 6.8 ms, field of view = 24 cm, 256 mm × 256 mm matrix, and isotropic 0.9 mm thick slices), and a 3D pseudo-continuous ASL sequence with a single post-labeling delay (PLD) (labeling duration = 1450 ms; PLD = 2025 ms).

### Image analysis

A neuroradiologist and a senior trainee (37 and 5 years’ experience, respectively) simultaneously and collaboratively analyzed together all anonymized retrospectively obtained images on a Sectra PACS review workstation (Linköping, Sweden). ASL + was defined as focal ASL signal hyperintensity within the JB venous lumen, with or without distal sigmoid sinus involvement, confirmed by direct anatomical co-localization on post-contrast SPGR images. We classified ASL + as present or absent based on visual assessment relative to adjacent venous and background signal, with exclusion of arterial signal based on anatomical location. We used click-and-drag electronic calipers in the PACS built-in image measurement tools to obtain all morphometric measurements on digital post-contrast SPGR volumetric images that optimally showed the JB and its venous tributaries. Any minor dissimilarities arising between raters (notably, there was an excellent reliability coefficient of > 0.90 for our first-time agreements) as they jointly performed the quantitative, objective/unambiguous measurements, were discussed and settled by additional consensus agreement.

### Data collection

We recorded patient demographics and IJV dominance. As stated, we did not evaluate right-sided JB morphometrics because this ASL artifact rarely occurs on that side; we therefore compared findings in patients with and without artifact on the left only. On post-contrast SPGR images for both cohorts, we measured: (1) diameters of distal sigmoid sinuses, inferior petrosal sinuses, and PCVs, all at vessel confluences with the JBs; and (2) maximum JB axial areas. We then used reformatted parasagittal images to measure: (3) dimensions of narrowest points of inflow, midportion, and outflow of each JB; and (4) any presence of JB diverticula. Lastly, we obtained (5) 3D volumes of JBs after manual segmentations in orthogonal planes.

### Statistical analysis

We performed inter-group comparisons of independent means using a *t*-test to study associations between measurements of JBs and tributaries, and presence or absence of ASL hyperintensity; and Pearson correlations for age associations. We also calculated Cohen’s d-effect sizes to characterize the magnitude of between-group differences. Of note, we anticipated if PCVs (the smallest measured veins) were found to be in the size range of two to three times the spatial resolution on 0.9-mm isotropic post-contrast SPGR imaging, their diameter measurements would be treated as exploratory and interpreted with awareness of potential partial-volume and plane selection effects. Statistical significance was set at *P* < 0.05.

#### Univariate ROC analysis

To investigate the feasibility of establishing practical clinical guidelines to distinguish ASL+ status using JB dimensions as potential imaging discriminators, we evaluated accuracy of a logistic regression statistical model in distinguishing presence of ASL+ using three JB and tributary dimensions found to be significantly smaller in ASL+ than in CSs (see Results): the mean diameter of the JB midportion when viewed parasagittally, mean diameter of the narrowest point of JB outflow when viewed parasagittally, and mean diameter of PCVs. For this, we constructed receiver-operating-characteristic (ROC) curves for these three dimensions using Python (Python Software Foundation, Wilmington, DE, USA). Since a smaller venous diameter correlated with ASL+, we negated the measurement to ensure the binary classification would work as expected, with ASL+ indicating the positive class. We calculated area-under-curve (AUC) and cutoff values providing optimal balance between sensitivity and specificity, as determined by Youden’s index. We then used linear interpolation to estimate cutoff and specificity values not directly available in the data for when sensitivity was set at 90%, that is, what would be considered optimal for a diagnostic biomarker.

#### Multivariate ROC analysis

Next, we performed multivariate ROC analysis using logistic regression to evaluate the discriminatory potential of combining all three venous dimensions found to be significantly smaller in ASL+ than in CSs. This approach allowed us to assess the combined diagnostic value of all three measurements at once, potentially capturing important information that individual measurements might overlook. We first standardized the measurements with z-score normalization. We then took a 3-dimensional point representing all three measurements and transformed it to a single number using a logistic regression model to establish the probability of ASL+ (*P(ASL+)*), as:

$$P\left(ASL+\right)=\frac{1}{1+{e}^{-L}}$$

where L = β₀ + β₁x₁ + β₂x₂ + β₃x₃, and each *β* is a tunable parameter: *β₀* is a bias term, *β₁*, *β₂*, and *β₃* are correlation coefficients; and *x*_*1*_, *x*_*2*_, *and x*_*3*_ are the standardized z-score values for JB midpoint (M), JB outflow (O), and posterior condylar (PC) vein diameters. Thus, we derived:

$$L=-0.0304-0.0557\frac{\left(M-10.08\right)}{3.37}-0.6261\frac{\left(O-8.75\right)}{2.90}-1.0220\frac{(PC-2.54)}{0.87}$$

The multivariate model was then trained on the same 50 patients (25 ASL + and 25 CSs) as the individual ROC analyses to classify ASL+ status probability based on the combination of the three measurements. We generated a composite ROC curve from the established probabilities and calculated the AUC to assess model performance. We used linear interpolation to estimate sensitivity and specificity values at precise threshold points not directly available in the data.

To assess potential overfitting of both univariate and multivariate models, we performed leave-one-out cross-validation (LOOCV), in which each patient was iteratively held out as a test case while the model was trained on the remaining 49 patients. Cross-validated AUC values were calculated from the aggregated predicted probabilities across all 50 iterations. To address the potential of false-positive findings from multiple comparisons across the eight morphometric variables tested, we applied Benjamini-Hochberg false discovery rate (FDR) correction and permutation testing for 10,000 permutations.

Additionally, we explored thresholds for different sensitivity and specificity levels using the Streamlit^®^ app (Snowflake Inc., Bozeman, MT, USA), a cloud-based platform for uploading our Python scripts, running them in a web browser, and sharing the visualizations publicly at https://asl-roc.streamlit.app.

We also used a nonparametric DeLong test to statistically compare the combined AUC value from multivariate ROC analysis with AUC values obtained for individual venous measurements from univariate ROC analysis.

## Data Availability

From the corresponding author upon reasonable request.
